# A Technology-Enriched Approach to Studying Microlongitudinal Aging Among Adults Aged 18 to 85 Years: Protocol for the Labs Without Walls Study

**DOI:** 10.2196/47053

**Published:** 2023-07-06

**Authors:** Brooke Brady, Shally Zhou, Daniel Ashworth, Lidan Zheng, Ranmalee Eramudugolla, Md Hamidul Huque, Kaarin Jane Anstey

**Affiliations:** 1 School of Psychology University of New South Wales Randwick Australia; 2 University of New South Wales Ageing Futures Institute University of New South Wales Sydney Australia; 3 Neuroscience Research Australia Sydney Australia

**Keywords:** life-course aging, longitudinal research, subjective age, gender, cognition, sensory function, app, mobile app, eHealth, mobile health, mHealth, measurement burst design, ecological momentary assessment, health information technology, personalized health, mobile phone

## Abstract

**Background:**

Traditional longitudinal aging research involves studying the same individuals over a long period, with measurement intervals typically several years apart. App-based studies have the potential to provide new insights into life-course aging by improving the accessibility, temporal specificity, and real-world integration of data collection. We developed a new research app for iOS named Labs Without Walls to facilitate the study of life-course aging. Combined with data collected using paired smartwatches, the app collects complex data including data from one-time surveys, daily diary surveys, repeated game-like cognitive and sensory tasks, and passive health and environmental data.

**Objective:**

The aim of this protocol is to describe the research design and methods of the Labs Without Walls study conducted between 2021 and 2023 in Australia.

**Methods:**

Overall, 240 Australian adults will be recruited, stratified by age group (18-25, 26-35, 36-45, 46-55, 56-65, 66-75, and 76-85 years) and sex at birth (male and female). Recruitment procedures include emails to university and community networks, as well as paid and unpaid social media advertisements. Participants will be invited to complete the study onboarding either in person or remotely. Participants who select face-to-face onboarding (n=approximately 40) will be invited to complete traditional in-person cognitive and sensory assessments to be cross-validated against their app-based counterparts. Participants will be sent an Apple Watch and headphones for use during the study period. Participants will provide informed consent within the app and then begin an 8-week study protocol, which includes scheduled surveys, cognitive and sensory tasks, and passive data collection using the app and a paired watch. At the conclusion of the study period, participants will be invited to rate the acceptability and usability of the study app and watch. We hypothesize that participants will be able to successfully provide e-consent, input survey data through the Labs Without Walls app, and have passive data collected over 8 weeks; participants will rate the app and watch as user-friendly and acceptable; the app will allow for the study of daily variability in self-perceptions of age and gender; and data will allow for the cross-validation of app- and laboratory-based cognitive and sensory tasks.

**Results:**

Recruitment began in May 2021, and data collection was completed in February 2023. The publication of preliminary results is anticipated in 2023.

**Conclusions:**

This study will provide evidence regarding the acceptability and usability of the research app and paired watch for studying life-course aging processes on multiple timescales. The feedback obtained will be used to improve future iterations of the app, explore preliminary evidence for intraindividual variability in self-perceptions of aging and gender expression across the life span, and explore the associations between performance on app-based cognitive and sensory tests and that on similar traditional cognitive and sensory tests.

**International Registered Report Identifier (IRRID):**

DERR1-10.2196/47053

## Introduction

### Background

Longitudinal aging research involves studying the same individuals over a long period, typically with measurement intervals several years apart. Although this approach can inform long-term developmental processes, it cannot capture processes that fluctuate on multiple—sometimes acute—microlongitudinal timescales. This project aims to contribute to the contemporary theories of life-course aging and gender by establishing a novel methodology for examining their daily experiences, known as the Labs Without Walls research app. We seek to evaluate the feasibility and usability of the app and a paired Apple Watch (Apple Inc) for studying the microlongitudinal processes of aging among a life-course sample of Australian adults. The validity of the app-based administration of common cognitive and sensory tasks will also be explored.

### Benefits of App-Based Approaches for Longitudinal Aging Research

Recent data suggest that 91% of Australians aged 18 to 75 years own a smartphone, and 58% use a smartphone or smartwatch device to monitor their health or well-being [1]. The rise in the popularity of smartphone devices has been accompanied by a commensurate rise in app-based studies of health and well-being. However, it is notable that many of these studies are designed for younger cohorts or specific clinical populations [2,3]. Few studies have examined processes that may differ across the life course [4].

App-based approaches to research offer a number of benefits for conducting longitudinal research [5] that may provide new insights into many factors that may be associated with developmental changes across the human life span, which we term *processes of life-course aging*. First and foremost, research apps remove the physical barriers to participation that are a feature of more traditional life-course aging studies. Apps can be accessed from anywhere with an internet connection, and this opens participation opportunities to adults living remotely or experiencing mobility restrictions that would otherwise prevent travel to urban research centers. Apps can be convenient for both participants and researchers, as they can allow participants to configure their testing and alert schedules to times that suit their daily routines and reduce researcher workloads associated with in-person testing sessions. Well-designed apps can also be interactive and engaging. This may be particularly important for studies that require participants to provide regular, intensive data.

Smartphones and paired wearable devices also include a range of sensors that provide new opportunities to noninvasively study complex microlongitudinal processes. For example, mobile apps can be designed to collect high-quality passive data (including physical activity metrics, heart rate, location data, and noise exposure), which can help researchers obtain more accurate and detailed data than possible using other methods. These data can be collected and integrated in real time, which can be useful for studying dynamic or rapidly changing phenomena and improving the ecological validity of research.

Finally, although substantial investment is often required in the development stage of bespoke research apps, app-based research can be a cost-effective option, as they can reduce the need for in-person research sessions and specialized equipment and leverage free-to-use open-source cognitive, sensory, and behavioral testing resources for research, such as those provided by the Apple ResearchKit framework (Apple Inc) [6].

### This Study: Capturing Day-to-Day Subjective Experiences of Age and Gender

Age and gender are 2 defining characteristics used in nearly all human studies. The perceptions and expressions of each are influenced by social, biological, and environmental determinants, and people’s subjective experiences of age and gender have been vastly understudied. Self-perceptions of aging (SPA) is a multidimensional construct involving the personal evaluation of age-related changes experienced in various life domains, irrespective of chronological age [7]. Subjective age is a form of SPA and refers to how young or old individuals experience themselves to be. SPA is associated with numerous health outcomes, including mortality [8], mental health [9], and functional health [10]. SPA changes over the life course. Previous research has shown that adolescents perceive themselves as older than their chronological age (age in years from the date of birth), potentially reflecting increased perceptions of maturity [11]. This changes in early adulthood, when people usually hold younger subjective ages. The discrepancy between the chronological and subjective age increases across the life span [11,12]. Life-course variability in SPA may reflect psychosocial changes experienced at different life stages; however, there is yet to be a study exploring SPA among a life-course sample. Despite strong evidence of long-term variability in SPA, limited data exist on how SPA fluctuate on microtimescales, that is, hours, days, or weeks. Existing day-to-day variability in measures of subjective age, an area of SPA, suggests fluctuations in multiple domains [13]. The measurement timescale may greatly alter the perceived relationship between variables [14] and could help elucidate intraindividual variability in SPA and the interplay between SPA and other time-varying constructs, such as mood, stress, and physical activity.

Gender, similar to many other social constructs, can change and evolve over time [15]. Gender is often conflated with sex, although they are distinct constructs. *Sex* commonly refers to a person’s biological status as male, female, or another variation of sex characteristics. Indicators of biological sex can include sex chromosomes (XX and XY), hormones, internal reproductive organs, and external genitalia [16]. In research practice, sex is usually reported as male or female based on the sex recorded at birth. *Gender* is a dynamic social construct that refers to the psychological, social, and cultural factors that shape attitudes, behaviors, stereotypes, and knowledge. In research practice, gender is typically treated as a fixed, binary variable despite substantial variations in the real world [17]. Such an approach does not take into account the multidimensional nature of gender, which includes gender norms (spoken and unspoken rules within the family, community, institution, or culture), gender relations (power relations between individuals with different gender roles and identities), gender identity (how individuals and groups perceive and present themselves within specific cultures), and gender expression (the outward expression of one’s gender identity, including physical and behavioral dimensions) [16], nor does such an approach allow for an understanding of gender as dynamic [15]. Indeed, gender dynamism can be seen in at least 3 aspects: the meaning of gender has changed over time; there are substantial cultural differences in the meaning of gender; and one’s own gender and relationship with it can change, evolve, weaken, and galvanize across the lifetime [15]. There is also likely to be heterogeneity in gender dynamism at the individual level and between and within age and sex-at-birth groups, which has not been the subject of study to this point.

Theoretical accounts of socioemotional aging suggest that intraindividual variability in gender expression may be greatest during early adulthood—when broad social networks and diverse social interactions are used to help craft an individual’s self-concept—and decrease at older ages when interactions with diverse identities are reduced and one’s sense of self is more clearly defined [18]. This study seeks to understand the factors that impact daily variability in gender expression across the life span and how variability may differ between male participants and female participants. Research has not explored the variability in SPA and gender expression and its relationships with both stable and variable personal, social, and environmental factors. Improving the understanding of life-course variability in self-perceptions may help identify complex relationships among self-perceptions, health, and the environment.

### Aims of This Study

Technology has enabled vast improvements in app-based research that have yet to be used to study SPA or gender expression across the life course. Therefore, the aim of this study is to evaluate the feasibility and usability of the newly developed Labs Without Walls research app and a paired Apple Watch for studying life-course aging among male and female participants from different age groups. We hope to disentangle processes that may vary across microtimescales, including SPA and gender expression. The study also aims to validate the app-based administration of common cognitive and sensory tasks for use among Australian life-course samples.

### Hypotheses

We hypothesize the following:

Hypothesis 1: Participants aged 18 to 85 years will be able to successfully provide e-consent, be able to input survey data through the Labs Without Walls research app, and have passive data collected using an Apple Watch over 8 weeks.

Hypothesis 2: The user experience of the Labs Without Walls research app and Apple Watch will be rated as acceptable by research participants aged 18 to 85 years.

Hypothesis 3: Data collected on multiple timescales using the Labs Without Walls research app will enable the exploration of variability in SPA and how SPA may be affected by personal characteristics, environmental conditions, physical health, resilience, fatigue, stress, and mood.

Hypothesis 4: Data collected using the Labs Without Walls research app will also allow for the exploration of stability and variability in gender expression and how gender expression may be affected by personal characteristics, physical health, resilience, mood, stress, and fatigue.

Hypothesis 5: Cognitive and sensory assessments delivered through the Labs Without Walls research app will be comparable with the traditional face-to-face versions of the assessments.

## Methods

### Participants

We aim to recruit a total of 240 Australian adults stratified by sex (male and female) and age group (18-25, 26-35, 36-45, 46-55, 56-65, 66-75, and 76-85 years).

#### Power

Sample size calculations for the full sample and the face-to-face validation subsample were estimated using the following criteria: threshold probability for rejecting the null hypothesis (2-tailed =.05) and the probability of failing to reject the null hypothesis under the alternative hypothesis (=.20). This pilot study is exploratory in nature. However, based on a previous meta-analysis [19], the odds ratio of subjective age impacting overall health is estimated to be 1.57. Using the aforementioned criteria, G*Power [20] was used to calculate the minimum sample size of 129 participants for the full study, which was well below our target. To allow for the addition of multiple covariates in statistical models and potential cases of missing data or attrition across the 8-week study period, we aim to recruit a total sample of 240 participants.

For the face-to-face validation subsample, a minimum sample size of 29 participants is required to detect a moderate correlation of *r*=0.50 between the performance on app-based cognitive and sensory tasks and that on face-to-face cognitive and sensory tasks, based on α (2-tailed)=.05 and β=.20 [21]. We aim to oversample to a total of 40 participants to account for possible missing data or the withdrawal of consent across the study.

#### Recruitment

Between May 2021 and January 2023, research volunteers will be recruited from the general Australian population using email, social and professional media call outs, and the Neuroscience Research Australia (NeuRA) Healthy Research Volunteer Registry. Participants will be invited to read the study information on the web via the study website. Web-based recruitment materials include a link to the study website, where those who are interested enter their contact details and preferred method of contact (phone, email, or text) for the research team to conduct eligibility screening.

#### Eligibility

To be eligible to participate in this study, adult volunteers must be living in Australia, be aged 18 to 85 years, be a current iPhone (Apple Inc) user, be willing to use their iPhone to download free research apps, be proficient in English, be able to access a home Wi-Fi connection for the duration of the study, not be reliant on text-to-speech services to use their iPhone, be willing to wear the Apple Watch during the study period and use headphones to complete hearing tasks, be willing to provide informed consent and comply with the study protocol, and agree to return the loaned study equipment (Apple Watch and headphones) at the end of the study.

As noted earlier, approximately 40 participants who join the study and are located in metropolitan Sydney will be invited to attend a 2-hour onboarding session either at the NeuRA or their homes to complete the traditional face-to-face versions of cognitive and sensory measures to assess validity across face-to-face and app-based administration methods.

### Materials and Devices

#### iPhones

Study participants will use their own iPhones (model 6S or newer, supporting iOS 13 or later) in the study.

#### Apple Watches

Participants can join the study with their own Apple Watch or can elect for the study team to post them an Apple Watch Series 5 GPS for use during the study period. In this study, Apple Watches will be used to collect a range of health and behavioral data (as outlined in Table 1). In addition, Apple Watches will be used to deliver task reminders to the participants.

**Table 1:** Brief description of the app- and laboratory-based cognitive and sensory tasks administered in the Labs Without Walls Study.

Format and task name		Source	Expected duration (minutes)	Construct or constructs measured	Description
**App-based testing**					
	Trail Making Task A	[6]	2	Sequencing, visual search, and processing speed	The participant uses their finger to connect a series of labeled circles presented on their phone screen, in order. The circles are labeled with sequential numbers (1, 2, 3, etc).
	Trail Making Task B	[6]	2	Cognitive flexibility, alternating attention, sequencing, visual search, and processing speed	The participant uses their finger to connect a series of labeled circles presented on their phone screen, in order. The circles are labeled with alternating numbers and letters (1, a, 2, b, 3, c, etc).
	Spatial Memory Task	[6]	2	Visuospatial memory and executive function	The participant is asked to observe and then recall pattern sequences of increasing length in a game-like environment.
	Stroop Task	[6]	3	Cognitive flexibility and inhibitory control	The participant is shown a series of words that are displayed in color and must select the first letter of the color’s name.
	Tapping Speed	[6]	2	Motor function, tapping speed, tapping accuracy, and tapping rhythm	The participant rapidly alternates between tapping 2 targets on the touch screen. The task is repeated for the left and right hands.
	Tower of Hanoi	[6]	2	Problem solving	The participant is asked to solve a puzzle in a minimum number of moves. To solve the puzzle, the participant must move the entire stack to the highlighted platform in as few moves as possible.
	Reaction Time	[6]	2	Reaction time	The participant shakes their iPhone (Apple Inc) in response to a visual cue on the screen.
	9-Hole Peg Test	[6]	2	Hand dexterity	The participant uses 2 fingers to touch on an on-screen peg and drag it into an on-screen hole and across a line. Both hands are tested.
	dB HL Tone Audiometry task	[6]	6	Hearing ability	The participant listens through ear pods (provided by the research team) to a series of tones and taps the left or right button on the screen when they hear each tone. These tones are of different audio frequencies and play on different channels (left and right), with the volume being progressively increased until the user taps one of the buttons.
	Amsler Grid	[6]	1	Visual problems, such as macular degeneration	The participant observes an on-screen grid while closing 1 eye for any anomalies and marks the areas that appear distorted using their finger.
	16-Plate Ishihara Colour Deficiency Test	Adapted for mobile from the original 25-plate Ishihara colour deficiency test [22]	3	Red-green color deficiency	The participant is shown 16 images depicting a circle made of smaller red and green dots. Each circle is presented on a single screen. Within each circle, numbers appear in alternating colors. The participant must enter the number shown within each circle using an on-screen keyboard.
**Face-to-face testing**					
	Trail making task A	[23]	3	Sequencing, visual search, and processing speed	The participant is presented with a sheet of paper that contains numbers randomly scattered throughout. The task is to connect the numbers in sequential order as quickly as possible.
	Trail Making Task B	[23]	5	Cognitive flexibility, alternating attention, sequencing, visual search, and processing speed	The participant is presented with a sheet of paper that contains both numbers and letters randomly scattered throughout. The task is to connect the numbers and letters in alternating, sequential order as quickly as possible. For example, the participant would connect 1 to A, then A to 2, then 2 to B, and so on.
	WMS-III^a^ Spatial Span (forward span)	[24]	5-10	Visuospatial memory	The participant is shown of a series of spatial locations, typically marked by a set of blocks or cubes, arranged in a particular order on a board or table. The examiner then points to each location in sequence, and the participant being tested is required to touch or point to each location in the same order.
	Victoria Stroop Task	[25]	5-10	Cognitive flexibility and inhibitory control	The participant is asked to read aloud a list of color names, such as “red,” “green,” and “blue,” as quickly and accurately as possible. The participant is then presented with a list of color words that are printed in an incongruent color, such as the word “red” printed in blue ink and is instructed to name the color of the ink, not the word itself, as quickly and accurately as possible.
	SHOEBOX dB HL Tone Audiometry Test	[26]	10-20	Hearing ability	The participant wears headphones. A series of pure tone sounds are presented at different frequencies and loudness levels, and the participant is asked to respond each time they hear the sound. The test typically begins at a frequency of 1000 Hz and progresses to higher and lower frequencies.
	25-plate Ishihara Colour Deficiency Test	[22]	5-10	Red-green color deficiency	The participant is shown a series of plates with colored dots or patterns arranged in a specific way to form a number. The participant must identify the number on each plate.

^a^WMS-III: Wechsler Memory Scale-III.

#### Headphones

Participants will be posted a new pair of Apple in-ear wired EarPods with a lightning connector (model number MMTN2FE/A) for use during hearing tasks.

#### Labs Without Walls Research App for iOS

Labs Without Walls was developed to provide a means of conducting life-course aging research outside traditional research laboratories. It was conceived and developed by the investigators. The app is primarily built in Swift (Apple Inc), using XCode (Apple Inc) as the development environment. Objective-C (Apple Inc) is used for enhancements to the Apple ResearchKit framework [6]. Apple’s ResearchKit is used to provide functionality, including consent, questionnaires, prebuilt active tasks, and graphs. The app uses read-only access to the local health store on the participant’s iPhone via Apple’s HealthKit interface, subject to the participant’s consent. Data will be collected continuously throughout the study period from both the worn Apple Watch and the participant’s iPhone. The app fully supports light and dark mode presentations throughout.

#### Backend

A backend, hosted on the Amazon Web Services (AWS) platform, will be used to collect study data securely. The AWS offers a range of security measures to protect data stored in the cloud, including industry-standard encryption algorithms to protect data in transit and at rest. Data cannot be accessed without the appropriate decryption key, and access is restricted to only essential research staff approved by the ethics committee. Two-factor authentication will be implemented, and 6-month audits of data access will be conducted.

### Technical Architecture

**Figure 1:**
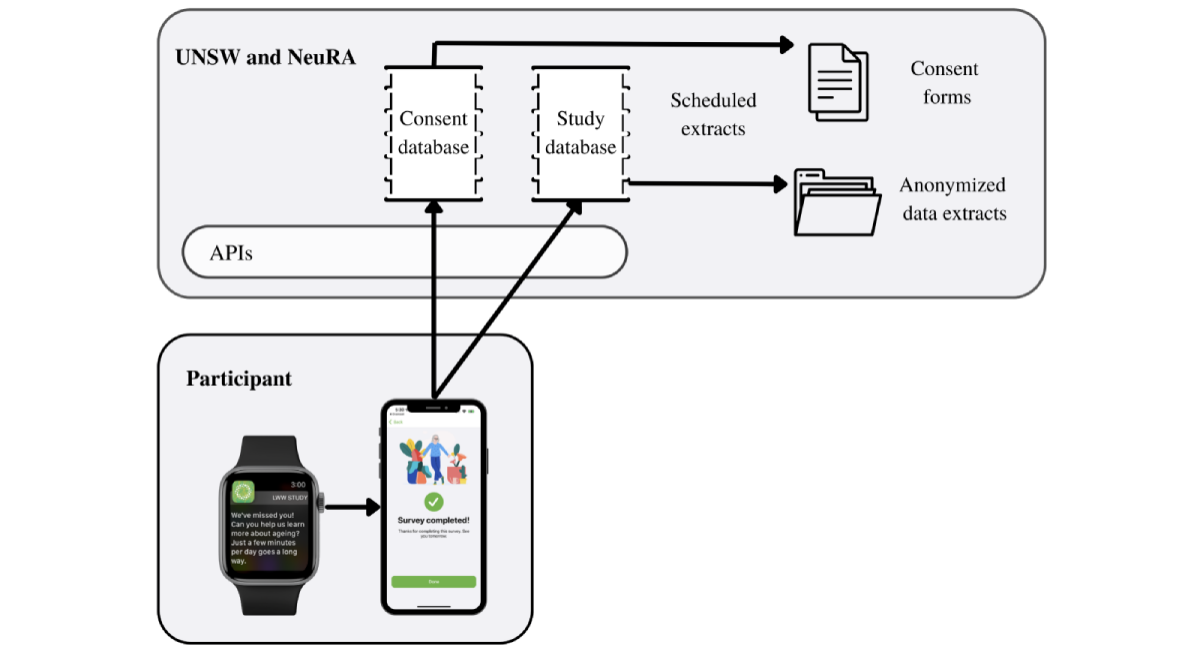
Technical architecture of the Labs Without Walls research study. API: application programming interface; NeuRA: Neuroscience Research Australia; UNSW: University of New South Wales.

The technical architecture of the Labs Without Walls study is summarized in Figure 1.

The technical architecture of the app is designed to separate consent forms and other identifiable information from deidentified study data. The consent details provided, including those in the signed e-consent form, will be transmitted by secured application programming interfaces to an AWS backend, where they will be stored in the stand-alone consent database. All other study data collected from participants (including data from questionnaires and cognitive and sensory tasks and health data) will be initially stored on the iPhone and transmitted to the backend when the app is running and has an active internet connection.

Two sets of data extracts will occur on a periodic basis. First, consent forms will be extracted to a secured location (S3 bucket), accessible to a limited number of members of the study team. These will be retained as a record of informed consent. Second, the collected study data will be extracted for analysis. These extracts will be anonymized to remove personally identifying information. These will be extracted to a separate secured location (S3 bucket) and will be accessible only to a limited number of members of the study team. Data extracts will be stored and handled according to an approved University of New South Wales Research Data Management Plan.

### Procedure and Study Design

**Figure 2:**
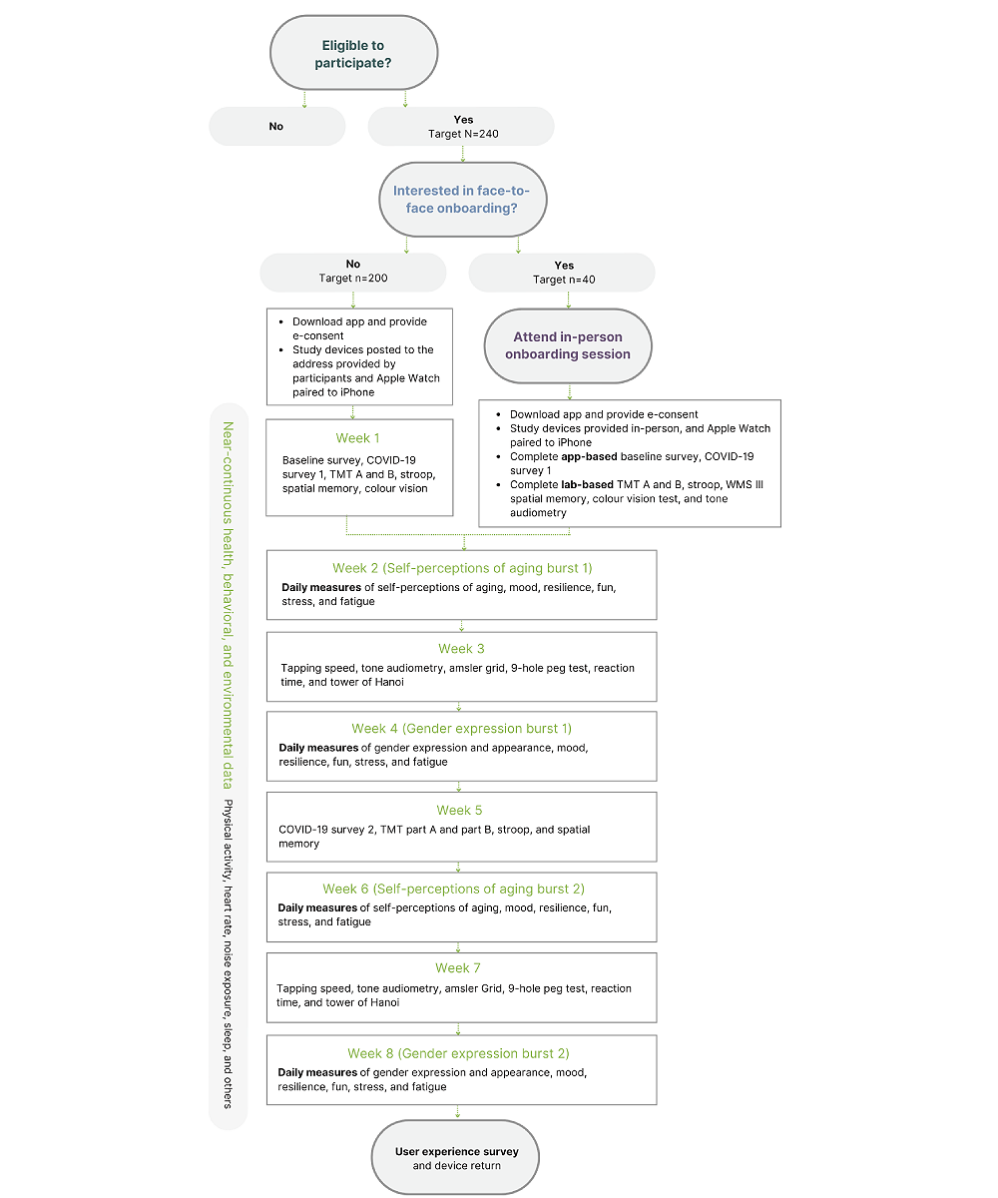
Flowchart summarizing the Labs Without Walls study procedure from eligibility screening to user experience feedback. TMT: Trail Making Task; WMS-III: Wechsler Memory Scale-III.

This project uses a multitimescale measurement burst design [27,28] to collect single–time point, near-continuous, and repeated-measure data from participants using the Labs Without Walls research app and an Apple Watch. Participants will complete a total of 31 short surveys and 14 active tasks using the research app. The completion of tasks is staggered across the 8-week period. The study procedure is summarized in Figure 2. The time commitment for each day will not exceed an average of 5 to 10 minutes. At the end of the study, after completing a user satisfaction questionnaire, participants will be asked to return any borrowed devices (Apple Watch and headphones) using a supplied prepaid mailing box. Participants may also choose to leave the study early from within the app and specify whether they consent to the data previously collected from them being used in the study. Participants will not receive payment for joining the study. However, participants who attend the NeuRA for face-to-face cognitive and sensory testing will be offered a voucher worth Aus $20 (US $13) to assist with travel costs.

### Ethics Approval

Ethics approval was obtained from the University of New South Wales Human Research Ethics Committee (HC200792).

### Participant App Experience

#### Joining the Study

Eligible participants will be given a link to download the Labs Without Walls app from the Apple’s public App Store and install it onto their existing iPhone. Alternatively, as the app is publicly visible in Australia, participants can search for it using the App Store app on their device. Each participant will be issued a unique 1-time invitation code to allow them to join the study within the app. Once their invitation code is accepted, the user can proceed to the study consent process within the app.

#### e-Consent

The e-consent process obtains and records the consent of the participant within the app before they participate in the study.

**Figure 3:**
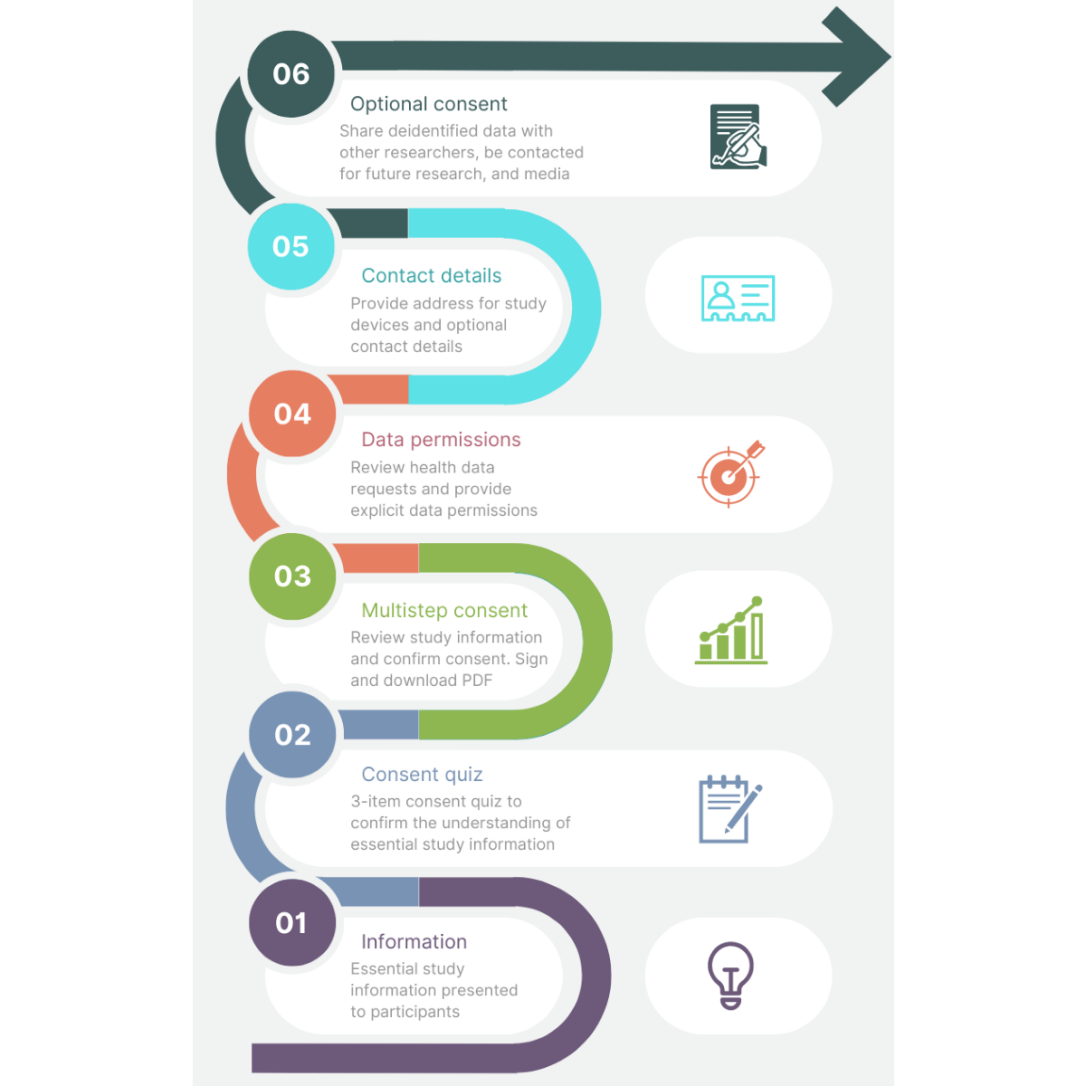
Summary of the Labs Without Walls in-app consent flow.

The Labs Without Walls e-consent flow, summarized in Figure 3, is presented in Textbox 1.

**Labs Without Walls e-consent flow.**

- An introductory page will appear.
- Multiple pages presenting essential study information will appear.
- A consent quiz to check the participant’s understanding of key consent concepts will be administered. The participant needs to complete the quiz correctly to proceed (can retry as required).
- A review page with an Agree button, which the participant can tap on to proceed further, will appear.
- When the participant taps on Agree, a confirmation message will be shown.
- If the participant confirms their agreement, then they will be taken to a signature capture page, to obtain their signature.
- A page where the participant can allow access to their health data and choose which measures are to be accessible (refer to the *Health Data Collection* section) will appear.
- Contact information will be collected to allow for device mail-out.
- Pages where the participant can provide optional consent for the use of anonymous data by other approved researchers, contact for further studies, and contact for media events will appear.
- A completion page will appear.

Once consented, participants will be posted an Apple Watch (if not joining with their own device) and Apple EarPods for use during the study. Once participants pair the watch with their phones, they will begin the 8-week study period.

#### Study Tasks

##### Task List

At any time, the list of available tasks to complete will be displayed on the home tasks screen. Tasks will remain in the task list for 3 days after the scheduled date, and the date of completion will be recorded in the data file. Tasks beginning the following day will also be shown in the task list, although not accessible until the scheduled date. Tapping on a task will start the task. A task can be a survey, cognitive task, or physical or sensory task. These are described in the subsequent section.

##### Survey Measures

**Figure 4:**
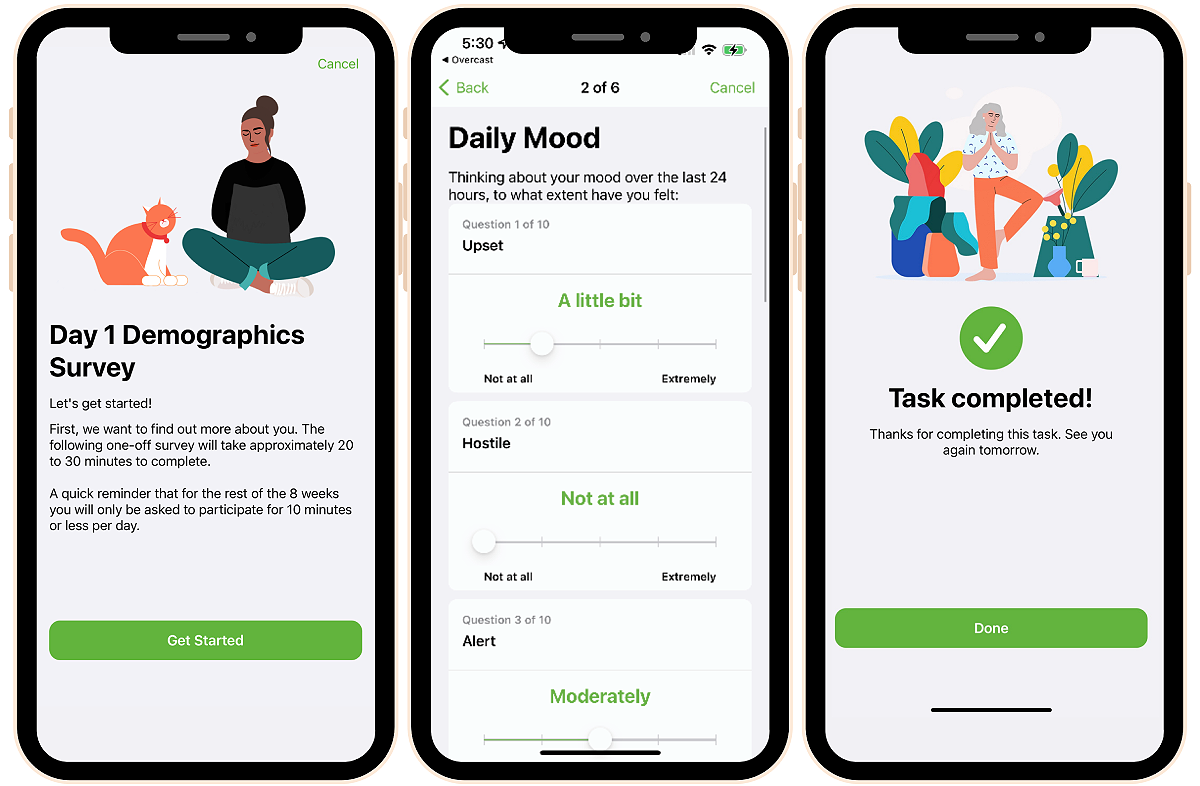
Sample introductory page, survey page with slider response options and completion page from the Labs Without Walls research app.

Much of the study data will be collected from participants in the form of short surveys. The Labs Without Walls research app includes one-off surveys (eg, baseline), surveys administered twice (COVID-19 experiences), and daily diary–style repeated-measure surveys (eg, SPA, gender expression, mood, and resilience). A list of the in-app surveys and their measurement frequencies is provided in Table 2. Composite scores—which can be automatically computed within the app—will be created according to the published guidelines for existing measures. The typical survey flow consists of an introductory page, multiple pages of questions, and a completion page (Figure 4).

**Table 2:** Surveys included in the Labs Without Walls research app and their measurement frequencies.

Construct of interest	Measure description	Items, n	Administration day or days
Age	Years	1	1
Ethnicity	Ethnic group or groups identified with	1	1
Relationship status	Married, in a relationship, or single	1	1
Education	Years of education by education stage (primary, secondary, technical college, university, or other)	5	1
Household income	Weekly average household income before tax	1	1
Employment	Employed, unemployed, or retired	1	1
Self-rated health	Physical and mental health measured by the SF-12^a^ version 2 [29]	12	1
Sex	Self-reported sex at birth	1	1
Gender identity	Self-reported gender identity	1	1
Childhood gender role nonconformity	Subset of items from the Recalled Childhood Gender Identity and Gender Role Questionnaire [30]	3	1
Genders in childhood home	Balance of genders in childhood home	1	1
Sexuality	Sexual orientation	1	1
Menopause	Self-reported female reproductive status (administered only to those assigned female at birth)	1	1
HRTs^b^	Currently taking HRTs	1	1
Smoking	Currently smoke	1	1
Vaping	Currently vape	1	1
Alcohol	Frequency of consumption and amount per occasion	2	1
Resilience	CD-RISC2^c^ [31]	2	1
Chemical intolerance	BREESI^d^ [32]	3	1
Perceptions of air quality	Perceived in-home air quality and perceived impact of quality on health	2	1
Future time perspective	Short-form FTP^e^ scale [33,34]	5	1
Expectations regarding aging	ERA-12^f^ [35]	12	1
Social isolation	UCLA^g^ Loneliness Scale [36]	3	1
Digital technology use	Weekday use of digital and internet technologies and impact of use	6	1
COVID-19 experiences	COVID-19 experiences and impact questionnaire	37	4 and 30
Subjective aging (daily)	Felt age, look age, behave age, mental performance age, interests age, and ideal age	6	1, 8-14, and 36-42
Mood (daily)	Adapted 10-item Positive and Negative Affect Scales [37]	10	8-14, 22-28, 36-42, and 50-56
Resilience (daily)	Adapted CD-RISC2	2	8-14, 22-28, 36-42, and 50-56
Fun (daily)	Original items measuring fun, laughter, and excitement in the past 24 hours	3	8-14, 22-28, 36-42, and 50-56
Gender expression (daily)	Gender appearance and expression adapted from the study by Wylie et al [38]	2	22-28, and 50-56
Stress (daily)	Original items measuring daily stress about work, finances, relationships, global events, and COVID-19	5	8-14, 22-28, 36-42, and 50-56
Fatigue and vitality (daily)	SF-36^h^ vitality subscale [39]	4	8-14, 22-28, 36-42, and 50-56
User satisfaction	Multifaceted feedback on the app and watch user experience	9	56

^a^SF-12: 12-Item Short Form Survey.

^b^HRT: hormone replacement therapy.

^c^CD-RISC2: 2-item Connor-Davidson Resilience Scale.

^d^BREESI: Brief Environmental Exposure and Sensitivity Inventory.

^e^FTP: Future Time Perspective.

^f^ERA-12: 12-item Expectations Regarding Aging Scale.

^g^UCLA: University of California, Los Angeles.

^h^SF-36: 36-Item Short Form Survey.

##### Feasibility and Acceptability Data

The feasibility and acceptability of the app and paired Apple Watch will be assessed in a number of ways. First, we will look at the number of successful consents—including successful completion of the 3-item consent quiz—as an indicator that the e-consent process is clear and reliable. The study retention rate will be examined as an indicator of how many participants remained in the study for the entire 56 days. Survey and task completion rates will be examined to look for potentially systematic gaps in the data or frequently incomplete surveys or tasks across participants. Participant-reported acceptability of the app will be assessed using 3 items: “Was the assessment schedule *manageable* in the context of your daily life? (Yes or No),” “How *user-friendly* did you find the mobile technology? (1=Not at all to 5=Extremely),” and “How much did you *enjoy* using the mobile technology? (1=Not at all to 5=Extremely).” Participants will be asked to provide feedback on alert frequency by responding to the item “What do you think about the frequency of alerts?” using a 9-point scale ranging from “too few” (1) to “just right” (5) to “too many” (9). Participant-reported acceptability of the watch as a study device will be assessed using 4 items: “How difficult did you find the watch setup process? (1=Not at all to 5=Extremely),” “How much did you enjoy wearing the watch? (1=Not at all to 5=Extremely),” “How comfortable did you find the Apple watch? (1=Not at all to 5=Extremely),” and “How difficult was it to charge the watch? (1=Not at all to 5=Extremely).” Participants will also be asked the question “What impact do you think the watch had on your physical activity?” to which they can respond using a 5-point scale ranging from “increased activity a lot” to “decreased activity a lot.” Participants will also be invited to provide general feedback by responding to the question “Do you have any other feedback on the research app that you would like to share with us?” using free text.

##### Cognitive Tasks

Several different cognitive tests will be conducted during the study, and in some cases, they will be repeated during the course of the study. App-based measures are a variety of active tasks, including the Trail Making Task (parts A and B), Spatial Memory, Stroop Test, Tapping Speed, Tower of Hanoi, Reaction Time, and 9-Hole Peg sourced from the Apple ResearchKit framework [6]. Laboratory-based cognitive measures include the Wechsler Memory Scale-III (WMS-III) Spatial Span (forward span) [24], Victoria Stroop Task [25], and the pen and paper version of the Trail Making Task (parts A and B) [23]. More information regarding the cognitive tasks is provided in Table 1.

##### Sensory Tasks

Several different sensory tests will be conducted during the course of the study, and in some cases, they will be repeated during the course of the study. We will use the dB HL Tone Audiometry task to assess hearing thresholds and the Amsler Grid to detect potential visual problems, sourced from the ResearchKit framework [6]. Our research team also developed an app-based version of the Ishihara Colour Deficiency Test [22]. Laboratory-based sensory tasks include a dB HL Tone Audiometry test administered using SHOEBOX Audiometry software (SHOEBOX Ltd) [26] and the 25-plate Ishihara Colour Deficiency Test. More information regarding the sensory tasks is provided in Table 1.

##### Alerts and Progress Reminders

**Figure 5:**
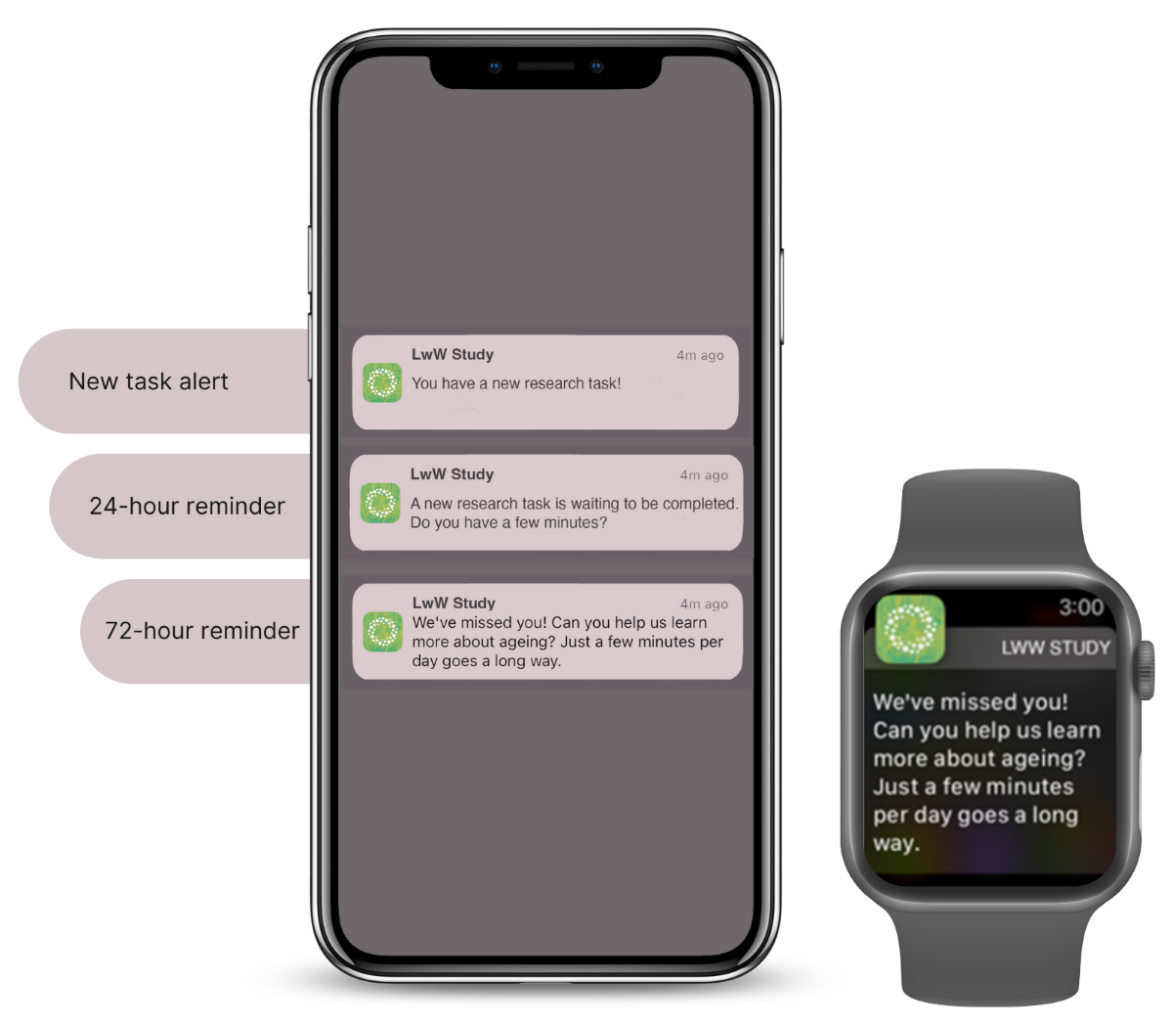
Sample task reminder alerts from the Labs Without Walls (LwW) research app on an iPhone and Apple Watch.

As new tasks are available for completion, notifications will be sent to the participant via both the iPhone and Apple Watch (subject to them allowing notifications on their phone). The notification time of day is user configurable in the app. Repeated notifications will be sent if the participant misses completing tasks for 1 day and 3 days, as shown in Figure 5. Tapping on a notification on their iPhone will take the participant to the task list page of the app. The task list page also includes a progress ring that displays the number of days completed and the number left to be completed. A balloon animation will be displayed to participants as they complete the project milestones (eg, week 1 complete and week 2 complete).

##### Health Data Collection

As noted, a range of health data measures will be collected throughout the study. These will be accessed from the health app on the participant’s iPhone, with their explicit on-device consent (see the *e-Consent* section). These data will be collected from the participant’s iPhone and a paired Apple Watch as appropriate (if worn). Participants can change their consent at any time using phone settings. Requested health measures are presented in Table 3.

Sleep data will be collected whenever the participant wears an Apple Watch while sleeping. For 1 week during the study, the participant will be asked to do so each night and will receive reminders to charge the watch in advance and put it back on before going to bed.

**Table 3:** Daily health, behavioral, and environmental data collected during the Labs Without Walls pilot study using an iPhone (Apple Inc) and an Apple Watch (Apple Inc).

Domain and measure			Input device		Are the data visible to participants in the app?
**Energy used**					
	Active energy (kJ)	Apple Watch		Yes	
	Basal energy (kJ)	Apple Watch		Yes	
**Activity**					
	Exercise (minutes)	Apple Watch		Yes	
	Stand time (minutes)	Apple Watch		Yes	
	Hours with at least 1 minute standing (number)	Apple Watch		Yes	
	Step count (number)	Apple Watch		Yes	
	Walk and run distance (km to 3 decimal places)	Apple Watch		Yes	
	Flights of stairs climbed (number)	Apple Watch		Yes	
	Hours the Apple Watch is worn (hour, to nearest quarter hour)	Apple Watch		Yes	
**Heart rate**					
	Resting (beats per minute)	Apple Watch		Yes	
	Walking (beats per minute)	Apple Watch		Yes	
	Maximum (beats per minute)	Apple Watch		Yes	
	Variability (SD of the interbeat [RR^a^] interval between normal heart beats)	Apple Watch		No	
**Mobility**					
	Walking speed (km per hour)	iPhone and Apple Watch		Yes	
	Walking step length (cm)	iPhone and Apple Watch		Yes	
	6-minute walk: meters (to a maximum of 500 m)	iPhone and Apple Watch		No	
	Stair speed (up and down; km per hour)	iPhone and Apple Watch		No	
	Gait—double support time (percentage of total walk time)	iPhone and Apple Watch		No	
	Gait—walking asymmetry (percentage of total walk time)	iPhone and Apple Watch		No	
**Noise exposure**					
	Environmental (average and maximum)	Apple Watch		Yes	
	Headphones (average and maximum)	iPhone		Yes	
**Sleep**					
	Hours slept (number)	Apple Watch		Yes	

^a^RR communicates the time between 2 successive heartbeats.

### Data Analysis Plan

#### Statistical Models

To answer hypothesis 1, aggregate data and summary statistics will be reported on the number of successful consents, retention rate, survey and task completion rates, app and watch usability, feedback on alert frequency, and hours of watch wear. To answer hypothesis 2, qualitative data on usability will be analyzed using thematic analysis, and quantitative data will be collected via Likert-type scales and analyzed using summary statistics (eg, mean and range). To answer hypotheses 3 and 4, the variability in SPA and gender will be analyzed using multilevel modeling. Random coefficient regression models will be used to analyze time-varying covariates. To answer hypothesis 5, correlation analysis will be used to establish the validity of the app-based versions of cognitive and sensory tests against laboratory-based tests. Log transformations will be used for any nonnormal data needed for parametric testing. In general, categorical variables will be coded numerically from low to high (eg, low=1 and high=5). The threshold probability for rejecting the null hypothesis will be set as (2-tailed)=.05, unless otherwise specified, and 95% CIs will be reported.

#### Data Exclusion and Missing Data

The data collected from the Apple Watch will be validated against the number of hours it has been worn each day. If the watch has been worn for <10 hours within a 24-hour period, data from that day will be excluded from the analyses. The average time taken to complete the surveys and tasks will also be checked for any outliers using box and whisker plots. Listwise or pairwise deletion or multiple imputation will be used to account for missing data where feasible and depending on what is most appropriate for the variables under consideration.

## Results

Recruitment began in May 2022. Data collection was completed in February 2023. The main project outputs will be (1) peer-reviewed publications, (2) conference presentations, and (3) research briefs that explain the study outcomes in a shorter format designed for a general audience. The publication of the preliminary results is anticipated in 2023.

## Discussion

### Expected Findings

There is a scarcity of studies that have examined the feasibility and acceptability of app- and smartwatch-based research technologies among life-course samples. This study aims to fill this gap by demonstrating the validity of these technologies among adults aged 18 to 85 years recruited from the general population of Australia. On the basis of previous research indicating high ownership and use rates of smartphones and smartwatches in Australia [1], it is expected that Labs Without Walls will prove to be acceptable, usable, and engaging for the life-course sample in this study.

This study aims to provide the first evidence for daily variability in multidimensional SPA from an Australian life-course perspective. SPA has been shown to be associated with mortality, mental health, and functional health [8-10]. Although long-term variability in SPA is well evidenced [11,12], far fewer studies have explored variability in SPA on shorter timescales. The findings of this study could shed light on intraindividual variability in SPA and the interplay between SPA and other time-varying constructs, such as mood, stress, and physical activity.

In addition, this study is expected to be the first to explore stability or variability in gender expression across time, as well as the degree to which gender expression is impacted by other factors such as age and sex at birth. In research practice, gender is typically treated as a fixed, binary variable despite significant variations in the real world [17], where gender dynamism can be seen in at least 3 aspects: the meaning of gender has changed over time; there are significant cultural differences in the meaning of gender; and one’s own gender and relationship with it can change, evolve, weaken, and galvanize across the lifetime [15]. This study aims to provide initial evidence exploring the degree of heterogeneity in gender dynamism at the individual level and between and within age and sex-at-birth groups.

The study also seeks to explore the cross-validity of app-based and laboratory-based administrations of common cognitive and sensory tests. Previous research has provided initial support for remote digital cognitive assessments in the context of cognitive aging [40] and psychiatric disorders [41]. The findings could add to the evidence base for remote digital cognitive assessments in the context of cognitive aging and psychiatric disorders.

Overall, the anticipated results of the Labs Without Walls study will inform the development of future iterations of the research app, aiming to answer ambitious questions regarding aging. We are interested in exploring the utility of other yet-to-be-used capabilities of the Apple Watch and iPhone in future studies. For example, GPS data collected using the Apple Watch or Apple smartphone can provide interesting insights into an individual’s “life-space,” which has been linked to well-being [42], mobility, and health [43,44]. Emerging behavioral and linguistic digital biomarkers of cognitive function are also promising [45,46]. By pilot-testing a Labs Without Walls approach, this study hopes to provide evidence for a platform that drives innovation in life-course aging research in Australia and beyond.

### Strengths and Limitations

This study has several limitations. Owing to budget restrictions, the Labs Without Walls app is built only for iPhones and is not available to Android users. Although iPhones are by far the most commonly used smartphones in Australia [47], we acknowledge that there is a selection bias inherent in the restricted availability of the app. We also acknowledge that the pilot version of the Labs Without Walls app does not include the functionality to enable adults with vision impairment to interact with it. In addition, app-based research can raise ethical concerns, such as the potential for privacy violations and the use of sensitive personal data. As detailed earlier, the research team has taken measures to protect the rights of participants, including their data security. Overall, the potential benefits of app-based approaches for conducting more accessible, convenient, and enjoyable research that also deliver more complex longitudinal data warrant dedicated efforts to develop apps that overcome (to the greatest extent possible) potential shortcomings.

### Conclusions

App-based studies have the potential to radically improve our understanding of processes that vary over microlongitudinal timescales. This study will provide evidence regarding the feasibility, acceptability, and usability of the Labs Without Walls research app and a paired Apple Watch for conducting research among a life-course sample of Australian adults. The feedback obtained will be used to improve future iterations of the app, explore preliminary evidence for intraindividual variability in SPA and gender expression across the adult life span, and explore the associations between the performance on app-based common cognitive and sensory tests and that on traditional common cognitive and sensory tests.

## Abbreviations

AWS: Amazon Web Services
NeuRA: Neuroscience Research Australia
SPA: self-perceptions of aging
WMS-III: Wechsler Memory Scale-III

## Data Availability

Study data sets and analytic codes will be deposited in publicly available repositories (where available and appropriate), as dictated by our funders, or presented in the main paper or additional supporting files whenever possible.
